# Functional and Molecular Effects of Arginine Butyrate and Prednisone on Muscle and Heart in the *mdx* Mouse Model of Duchenne Muscular Dystrophy

**DOI:** 10.1371/journal.pone.0011220

**Published:** 2010-06-21

**Authors:** Alfredo D. Guerron, Rashmi Rawat, Arpana Sali, Christopher F. Spurney, Emidio Pistilli, Hee-Jae Cha, Gouri S. Pandey, Ramkishore Gernapudi, Dwight Francia, Viken Farajian, Diana M. Escolar, Laura Bossi, Magali Becker, Patricia Zerr, Sabine de la Porte, Heather Gordish-Dressman, Terence Partridge, Eric P. Hoffman, Kanneboyina Nagaraju

**Affiliations:** 1 Center for Genetic Medicine Research, Children's National Medical Center, Department of Integrative Systems Biology, George Washington University School of Medicine and Health Sciences, Washington, D. C., United States of America; 2 Division of Cardiology, Children's National Medical Center, Washington, D. C., United States of America; 3 Pennsylvania Muscle Institute, University of Pennsylvania, Philadelphia, Pennsylvania, United States of America; 4 Department of Parasitology and Genetics, Kosin University College of Medicine, Pusan, South Korea; 5 Transgene SA, Illkirch-Graffenstaden, France; 6 Laboratoire de Neurobiologie Cellulaire et Moléculaire, Institut Fédératif de Neurobiologie Alfred Fessard, Cedex, France; National Institutes of Health, United States of America

## Abstract

**Background:**

The number of promising therapeutic interventions for Duchenne Muscular Dystrophy (DMD) is increasing rapidly. One of the proposed strategies is to use drugs that are known to act by multiple different mechanisms including inducing of homologous fetal form of adult genes, for example utrophin in place of dystrophin.

**Methodology/Principal Findings:**

In this study, we have treated *mdx* mice with arginine butyrate, prednisone, or a combination of arginine butyrate and prednisone for 6 months, beginning at 3 months of age, and have comprehensively evaluated the functional, biochemical, histological, and molecular effects of the treatments in this DMD model. Arginine butyrate treatment improved grip strength and decreased fibrosis in the gastrocnemius muscle, but did not produce significant improvement in muscle and cardiac histology, heart function, behavioral measurements, or serum creatine kinase levels. In contrast, 6 months of chronic continuous prednisone treatment resulted in deterioration in functional, histological, and biochemical measures. Arginine butyrate-treated mice gene expression profiling experiments revealed that several genes that control cell proliferation, growth and differentiation are differentially expressed consistent with its histone deacetylase inhibitory activity when compared to control (saline-treated) *mdx* mice. Prednisone and combination treated groups showed alterations in the expression of genes that control fibrosis, inflammation, myogenesis and atrophy.

**Conclusions/Significance:**

These data indicate that 6 months treatment with arginine butyrate can produce modest beneficial effects on dystrophic pathology in *mdx* mice by reducing fibrosis and promoting muscle function while chronic continuous treatment with prednisone showed deleterious effects to skeletal and cardiac muscle. Our results clearly indicate the usefulness of multiple assays systems to monitor both beneficial and toxic effects of drugs with broad range of *in vivo* activity.

## Introduction

Long-term development of definitive treatments for DMD, addressing the problem of the primary defect by gene supplementation, repair or compensation are all relatively time consuming processes, especially in the eyes of affected boys and their families. For this reason, there is considerable interest in testing potentially palliative agents that are currently registered for clinical application. Since such agents do not address the primary cause of the defect, and our understanding of the pathology downstream of this primary cause remains inadequate, they have to be judged empirically in terms of beneficial and deleterious effects. Candidates for this class of therapeutic agent are selected largely for their activities in other pathological conditions and our speculations as to other actions on what we suspect to be the main pathogenic pathways in DMD. Given the uncertainties involved, it is important to scan for indications of beneficial effect and, perhaps more importantly, for signs of deleterious side effects across as wide a range as possible of outcome measures. In the case of DMD, most primary investigations of putative therapeutic agents are conducted on the *mdx* mouse, because of its ease of production and maintenance, but most such studies concentrate on a small number of measures of outcome. In an attempt to remedy this, we have developed a protocol that encompasses a broad set of assessment criteria by which to detect subtle but significant effects of test agents on functional outcome measures in the *mdx* mouse, so arranged as to signal both desirable and undesirable effects of any given treatment regime [1]. Here we apply this protocol to a comparison of two different agents known or reputed to influence the course of the myopathy arising from dystrophin deficiency. First, prednisone which has well-recognized therapeutic value in DMD via as yet ill-understood mechanisms but which is associated with significant side effects that countermand its use in some individuals. Second, arginine butyrate, which has been postulated to exert beneficial effects via a spectrum of activities associated with the histone deacetylase, mediated control of expression of a variety of genes and also to provide a substrate for NOS signaling pathways. On this study we have used a wide variety of behavioral, functional, histological, biochemical, imaging and molecular assays to comprehensively assess the effects of these two drugs either alone or in combination in *mdx* mouse model of muscular dystrophy, We found that arginine butyrate treatment improved hindlimb grip strength and decrease fibrosis in the gastrocnemius but did not produce any significant improvements in muscle histology, behavioral measurements, or serum creatine kinase (CK) levels. In contrast, 6 months of chronic continuous treatment with prednisone as opposed to daily administration of a single dose resulted in deterioration in functional, histological, and biochemical measures. Our gene expression profiling experiments pointed to significant alterations in the expression of genes that control cell growth, differentiation, signaling, inflammation, and fibrosis in skeletal muscle as a result of arginine butyrate and prednisone treatment. Our results indicate the usefulness of multiple assays systems monitoring both beneficial and toxic effects of drugs with broad range of activity.

## Materials and Methods

### Animal Care

All mice were handled according to the local Institutional Animal Care and Use Committee guidelines. C57BL/10ScSn-Dmd*^mdx^*/J (*mdx*) and C57BL/10ScSn (BL10) female mice weighing 20–30 g (8- to 10-week-old) were purchased from The Jackson Laboratory (Bar Harbor, ME). All mice were housed in an individually vented cage system with a 12-h light-dark cycle and received standard mouse chow and water *ad libitum*. Mice were rested at least 10–14 days before acclimation was begun and baseline readings were obtained.

### Study design

The study involved four groups of 15 animals each: (a) a control group dosed with 0.9% NaCl (I.P) (the vehicle for the arginine butyrate preparation), (b) a group receiving arginine butyrate at 250 mg/kg/day I.P., (c) a group treated with prednisone at 1 mg/kg/day (slow-release subcutaneous pellets), and (d) a group (referred to as the combination-treated group) receiving arginine butyrate at 250 mg/kg/day I.P and prednisone at 1 mg/kg/day. Mice were treated for 6 months, beginning at 3 months of age for 5 days each week (Monday-Friday) for 6 months. The arginine butyrate solution was stored at room temperature. Prednisone slow-release subcutaneous tabs (Innovative America, Inc) are available as a 90-day release formulation with a daily slow release of 1 mg/kg. Two tabs were used per mouse, with the second tab being implanted after 3 months.

### Treadmill exercise

Mice were subjected to a 30-min run on a horizontal treadmill (Columbus Instruments, Columbus, OH) at 12 m/min to unmask the mild dystrophic phenotype. The test was performed during the morning hours twice weekly over the course of the 6 months, except on those days on which functional data were obtained.

### Rotarod test

Rotarod tests were performed as described previously [1], [2]: Mice were trained on the Rotarod (UgoBasile, VA, Italy) for 2 days before data collection. Each trial was done twice a day, with a 2-hour interval between sessions, for 3 consecutive days. The latency to fall (seconds) was recorded, and all six scores were averaged for each mouse. The averaged data were expressed as latency to fall (seconds) for each mouse.

### Grip strength test

Grip Strength was assessed using a grip strength meter consisting of horizontal forelimb mesh and an angled hindlimb mesh (Columbus Instruments, Columbus, OH). Five successful hindlimb and forelimb strength measurements within 2 minutes were recorded and normalized to body weight as previously described [1].

### Behavioral activity measurement

Open field activity was measured using an open field Digiscan apparatus (Omnitech Electronics, Columbus, OH) as described previously [1], [3]. All mice were acclimated for 60 minutes before actual data collection. Data were collected every 10 minutes over a 1-hour period each day for 4 consecutive days. Results were calculated as means±SE of all recordings.

### Echocardiography

Five mice per treatment group were scanned at 9 months of age. Mice were anesthetized with 1–2% isoflurane in 100% oxygen and scanning was performed over 20 minutes using a high frequency ultrasound probe (RMZ 702a, Vevo 660, VisualSonics, Toronto, Canada) as previously described [1], [4]. Qualitative and quantitative measurements were made offline using analytic software (VisualSonics, Toronto, Canada).

### Histological evaluations

At the end of the trial, all mice were euthanized, and tissue samples were taken for extensive testing as described below. A portion of each of the dissected muscles (e.g., gastrocnemius, diaphragm, and heart) was kept in formalin for H&E and Sirius Red staining. The remaining portion of each tissue was embedded in OCT compound and frozen in isopentane chilled in liquid nitrogen. Five non-overlapping representative fields of the tissue were imaged under a light microscope at an objective of 40× (high power field) and a digital image obtained using computer software (Olympus C.A.S.T. Stereology System, Olympus America Inc., Center Valley, PA). The digital images were loaded into Image J (NIH) with additional plug-in to count cells. Total number of cells, centralized nuclei, peripheral nuclei and total number of cells with centralized nuclei were counted and analyzed for comparison between treatment groups. Fibers showing degeneration (loss of striations/homogenous appearance of fiber contents) or regeneration (basophilic cytoplasm, large peripheral, or central nuclei with prominent nucleoli) and inflammatory foci per field were assessed in a blinded fashion as previously described [1].

### Fiber diameter measurements

Briefly, serial 8–10 µm thick frozen sections were cut using an IEC Minotome cryostat, mounted to super frost plus slides (Fisher Scientific) and fixed in cold acetone for 10 minutes. Sections were blocked using 100 µl 10% horse serum and incubated with primary Laminin antibody (rat anti ms Laminin). After two washes of PBS sections were applied with Goat anti-rat Alex Fluor 488 (Green). After two washes of 5 minutes with 1× TBS slides were mounted using Vectashield mounting medium with DAPI.

The tissue was imaged under a fluorescent microscope at an objective of 20×and a digital image obtained. The digital images were processed using Axiovision software and minimal feret's diameter was measured. To obtain the actual micron size we set a scale using a microscope scale (Graticules LTD Tonbridge, Kent, England) 100×0.01 = 1 mm. The data were ranked according to size, the ranks were then normalized to sample size (rank number/total sample number) and the normalized rank was plotted on the vertical axis against fiber size on the horizontal axis. Kolmogorov-Smirnov test was used to assess difference among fiber size distribution.

### Fibrosis measurements

Paraffin sections were stained with Sirius Red stain [Sigma-Aldrich, St. Louis, MO], and counter-stained with hematoxylin to visualize nuclei. The dye molecule intercalates into the tertiary groove in the structure of collagen types I and III and imparts a pink stain to most tissues when observed under white light. The tissue was imaged under a light microscope using a 4× objective, and a digital image was obtained using computer software (Olympus C.A.S.T. Stereology System, Olympus America Inc., Center Valley, PA). The digital images were processed using Image J (NIH), with an additional threshold color plug-in to process .jpeg images. The percentage of the fibrotic area corresponding to the area stained in red was compared to the total area of the tissue section, and the results were expressed as % non-muscle area.

### mRNA expression profiling

For RNA isolation, gastrocnemius tissue (from 4 mice/group, totaling 16 mice) was frozen in isopentane cooled with liquid nitrogen. The tissue was placed into a tube with 1 ml of Trizol and homogenized. The total RNA was isolated and then cleaned using the Qiagen RNeasy Mini kit (Qiagen, Valencia, CA) according to the manufacturer's instructions. The resulting total RNA was checked on a gel for RNA integrity and quantified using a Nanodrop ND-100 Spectrophotometer (Nanodrop Technologies, Wilmington, DE).

Gene expression profiling was carried out as described previously; using GeneChip mouse expression set 430 (Affymetrix 430 2.0, Santa Clara, CA) [5]. Probe set analysis was done using Microarray Suite, version 5.0. The signal intensity values (absolute analyses) of the probe sets were then loaded into GeneSpring (Silicon Genetics, Redwood City, CA) for further analysis. A *t*-test was performed to test the significance of any differences between the drug-treated and control group results.

### Western blotting

Total protein was extracted from frozen gastrocnemius muscle by placing the tissue in NP40 lysis buffer. Samples were run on a 4–12% BIS-TRIS 15-well gel (Invitrogen) for 2–2.5 h at 200 V and transferred onto a nitrocellulose membrane for 2 h at 300 mA. Following transfer, the blot was stained with Ponceau red to determine the efficiency of the transfer. The blot was then blocked in 5% milk for 1 h and incubated with anti-utrophin (1∶100) (NCL-DRP2, Novocastra) overnight at 4°C, washed five times for 6 min each with Tris buffered saline, and incubated with horseradish peroxidase (HRP)-conjugated goat anti-mouse IgG secondary antibody (1∶3000) for 1 h at room temperature. Following another series of washes with TBST, the blots were incubated for 1 min using ECL (Amersham), and the blots were developed. The blots were then stripped and blotted for beta-tubulin (55 kDa) to assess equal gel loading. The autoradiograms were scanned using an Arcus II scanner, and volume analysis was carried out using Quantity One software (Bio-Rad Discovery Series). The ratio of utrophin to beta-tubulin was calculated for each group of mice.

### CK determination

Blood was collected by heart puncture immediately after euthanasia with carbon dioxide: 250 µL of blood was collected into Eppendorf tubes with no additive for serum separation, and CK determination was performed using a standard spectrophotometric method. Assays were carried out using the enzyme-coupled assay reagent from Fisher Scientific (CK10) according the manufacturer's instructions. Absorption at 340 nm was measured every minute for 2 min at 37°C to calculate the enzyme activity. Duplicate measurements were done on each serum sample.

### Statistical analysis

Statistical analysis was performed by Student *t*-test to analyze the difference between two groups, One-way analysis of variance followed by Bonferroni post hoc test to compare the difference within groups and two-way analysis of variance to compare differences among groups and time. A total of eight histology measurements (degeneration fibers, regenerating fibers, inflammation, calcification, and central and peripheral nuclei) were compared between *mdx* and BL10 mice. Comparisons were made using Poisson regression or using negative binomial regression where the Poisson model did not fit the data due to over dispersion.

The Kolmogorov-Smirnov test for equality of distribution was used to compare 6 parameters between mice under different treatment conditions (saline treated, arginine-butyrate treatment, prednisone treatment, and a combination of prednisone and arginine-butyrate). Each treatment was compared in a pair-wise fashion and the resulting p-values were adjusted for multiple comparisons using the Sidak method. A nominal p-value of 0.05 was considered statistically significant. All analyses were done using Stata V10 (College Station, TX). The differences were considered significant at a p value of <0.05.

## Results

### Effect of 6 months treatment with arginine-butyrate, prednisone, or a combination of arginine butyrate and prednisone on *mdx* mice

#### Effect on body weight

**Figure 1:**
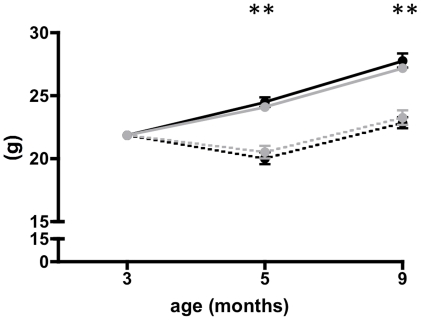
Effect of treatments on body weight. *mdx* mice received saline (solid black), arginine butyrate (solid grey), prednisone (broken black), a combination of arginine butyrate and prednisone (broken gray), for 6 months, beginning at 3 months of age. Body weights were obtained at 3 (baseline), 5, and 9 months of age. Error bars indicate +/− 1 SD. ** p = <0.001.

6 months administration of arginine butyrate to *mdx* mice did not significantly change their overall body weight and growth pattern when compared to saline-treated control mice (Figure 1). However, administration of prednisone or a combination of prednisone and arginine butyrate resulted in a significant decline in body weight during the first 2 months of treatment. The body weight gradually increased over the next 4 months, but was significantly lower than that of arginine butyrate- or saline-treated mice at 9 months of age (6 months of treatment; Figure 1). We then compared this data with age and sex matched BL10 wild type data that was previously published [1]. Saline and arginine butyrate treated *mdx* mice showed an increase of 103.12% and 100.93% respectively whereas prednisone and combination treated group displayed 84.7% and 86.33% of normal body weight respectively. This decrease in body weight was also reflected in a significant decrease in gastrocnemius muscle weight in the prednisone (absolute)- and combination-treated groups (absolute and normalized), than in arginine butyrate- and saline-treated groups (Supplementary Table S1).

No statistically significant differences were observed in the weight of heart tissue of the drug -and saline -treated groups but normalization showed significantly increased heart mass in prednisone and combination treated groups. The weight of soleus was significantly increased in arginine butyrate treated (absolute) group in comparison to saline treated group, however normalized values of soleus weights significantly increased in all treatment groups in comparison to saline treated group (Supplementary Table S1). We also observed significant decrease in spleen weights (absolute) in all three drug-treated groups with the exception of combination (normalized) group in comparison to the saline-treated mice (Supplementary Table S1).

#### Effect on behavioral measurements

**Figure 2:**
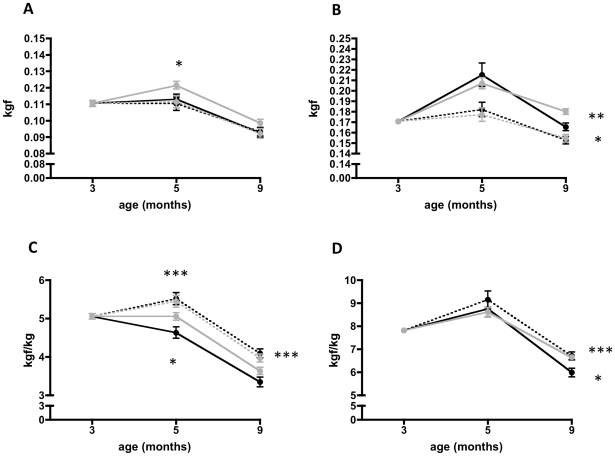
Effect of treatments on forelimb and hind limb grip strength. Grip strength was measured using a grid at 3, 5, and 9 months of age. a) Maximal forelimb grip strength, b) The maximal hind limb grip strength, c) The normalized maximal forelimb strength, d) The normalized maximal hind limb strength of all the groups. *Mdx* mice received saline (solid black), arginine butyrate (solid grey), prednisone (broken black), a combination of arginine butyrate and prednisone (broken gray), for 6 months, beginning at 3 months of age. Parameters were obtained at 3 (baseline), 5, and 9 months of age. Error bars indicate +/− 1 SD. * p = <0.01, ** p = <0.001, *** p = <0.0001.

Both the forelimb and hindlimb grip strength decreased in all groups by the end of treatment, at 9 months of age (Figure 2). The arginine butyrate treated group showed a significant improvement in unadjusted forelimb at 5 months compared to saline treated (0.12±0.01*vs.*0.11±0.01 p = 0.03). The rate of decline in the unadjusted forelimb strength was slower in the arginine butyrate-treated group than in the prednisone-, combination-, or saline-treated groups (Figure 2A). On the other hand hindlimb strength showed an increment from 3 months to 5 months and a subsequent decline at 9 months in saline and arginine butyrate treated animals, at which point arginine butyrate showed significant better strength than saline treated (0.16±0.01 *vs.* 0.18±0.01, p = 0.002). Prednisone and Combination treated groups showed a significant decline in hindlimb strength when compared to saline treated animals (p = 0.01, p = 0.04 respectively) (Figure 2B). However, normalization of forelimb strength with body weight revealed a slower decline in strength in the arginine butyrate-, prednisone-, and combination-treated groups than in the saline-treated group (Figure 2C). Again, arginine butyrate treated group showed significantly better strength than saline at 5 months (p = 0.01). The unadjusted hind limb strength was lower at 9 months of age in the prednisone- and combination-treated groups than in the arginine butyrate- or saline-treated groups. All the drug-treated mice appeared to show a slower decline than did the saline-treated control group at 9 months of age (Figure 2D), and arginine-butyrate treated group presented with a significant difference in strength at 9 months (5.98±0.67 *vs.*6.62±0.44 p = 0.004). We have compared this data with previously published BL10 wild type mice grip strength data and treatments resulted in 61.62% (Saline), 66.91% (Arginine butyrate), 75.32% (Prednisone) and 73.1%1 (Combination) change in forelimb grip strength and 65.16%% (Saline), 72.14% (Arginine butyrate), 72.99%% (Prednisone) and 72.42% (Combination) change with respect to BL10 wild type mice. Latency to fall (sec) on the Rotarod test is used to assess motor coordination and strength. All the treatment groups showed a decreased time on the Rotarod with increasing age (Supplementary Figure S1A). Similarly, in an analysis of behavioral measurements such as horizontal activity, vertical activity, and total distance using an open field activity (Digiscan) chamber, showed no statistically significant differences (Supplementary Figure S1B–1D).

#### Effect on histology

Examination of Hematoxylin and Eosin (H&E)-stained sections of gastrocnemius muscle of arginine butyrate-treated mice showed no significant change in total number of fibers, number of total central nuclei (refer to the amount of regeneration in a field) and number of fibers with central nuclei (fibers with centralized nuclei refers to cells undergoing regeneration cycle) number of degenerating fibers, regenerating fibers, peripheral nuclei, inflammatory loci and number of peripheral nuclei per high power field in comparison to saline treated group (Table 1).

**Table 1:** Comparison of histological parameters in the gastrocnemius muscle of treatments in mdx mice.

Histology measurement #	FP0023 (N = 8)	Combination (N = 7)	Prednisone (N = 8)	Untreated (N = 8)
	Mean±SEM	Mean±SEM	Mean±SEM	Mean±SEM
Number of fibers/field	78.61±6.62	70.90±6.08	69.06±8.24	69.27±5.05
Central Nuclei/field	54.31±3.81	46.19±3.79	46.56±3.76	46.61±3.01
Peripheral Nuclei/field	73.16±4.04	72.72±6.02	78.77±5.78	72.61±4.62
Number of fibers with Centralized nuclei/field	37.85±2.55	33.13±2.26	33.17±1.84	33.20±2.35
Degenerating fibers/field	8.25±2.84	10.25±2.53	11.38±2.41	6.50±1.71
Regenerating fibers/field	10.13±3.62	7.25±2.15	5.38±2.93	9.88±2.70
Inflammatory foci/field	2.13±0.85	2.38±1.95	3.25±1.52	1.13±0.55

# data was collected from 5 non-overlapping field from each sample and average of all samples within a group; * Analyzed as log (central/peripheral) using linear regression.

Since we noticed significant differences in body weight as well as muscle weight we decided to measure the fiber size distribution in all 4 groups of mice. The highest fiber size distribution (supplementary Figure S2) difference among treatment groups was found between 35 and 65 microns. Saline treated group showed the larger fiber sizes than prednisone and combination treated groups while arginine butyrate treated group showed an intermediate size between saline and prednisone or combination treated groups (Table 2).

**Table 2:** Effect of treatments on muscle fiber size: Kolmogorov-Smirnov equality-of-distributions test.

Group Comparison	P-value	P-value adjusted for multiple comparisons
Combination vs. AB	0.004	0.02
Combination vs. Prednisone	NS	NS
Combination vs. Saline	<0.001	0.001
AB vs. Prednisone	NS	NS
AB vs. Saline	0.03	NS
Prednisone vs. Saline	0.003	0.01

**Figure 3:**
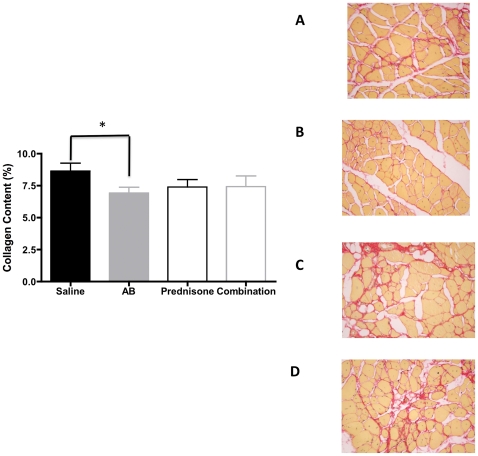
Effect of treatments on gastrocnemius collagen content. Sirius Red staining of formalin fixed gastrocnemius sections from four groups of mice. A representative picture of each group is shown (A–D). Saline treated (A), arginine butyrate treatment (B), prednisone- (C) and combination-treated groups (D). Quantitation of fibrosis (% fibrosis) was carried out using the ImageJ program.

**Figure 4:**
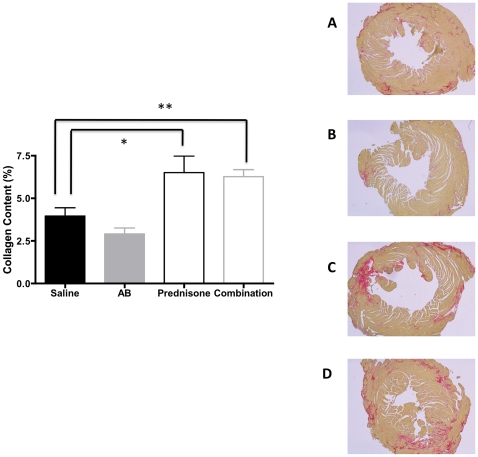
Effect of treatments on heart collagen content. Sirius Red staining of formalin fixed heart sections from four groups of mice. A representative picture of each group is shown (A–D). Saline treated (A), arginine butyrate treatment (B), prednisone- (C) and combination-treated groups (D). Quantitation of fibrosis (% fibrosis) was carried out using the ImageJ program.

**Figure 5:**
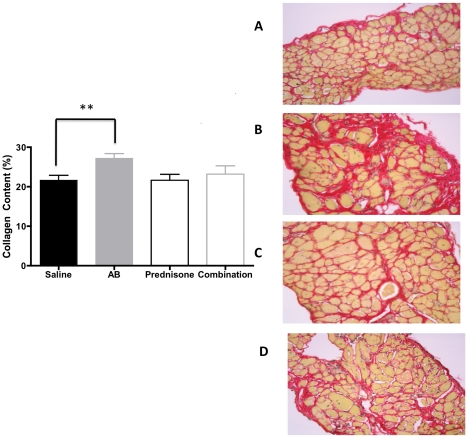
Effect of treatments on diaphragm collagen content. Sirius Red staining of formalin fixed diaphragm sections from four groups of mice. A representative picture of each group is shown (A–D). Saline treated (A), arginine butyrate treatment (B), prednisone- (C) and combination-treated groups (D). Quantitation of fibrosis (% fibrosis) was carried out using the ImageJ program.

We then further evaluated the heart, diaphragm and gastrocnemius muscle of these mice for fibrosis by staining the tissues with Sirius Red. Fibrosis was significantly decreased in gastrocnemius muscle of arginine treated mice (p = 0.04) compared to saline treated groups (Figure 3) There were no significant changes in collagen content of hearts in the arginine butyrate-treated hearts. However, the amount of fibrosis observed in the heart was significantly higher in the prednisone- and combination-treated groups compared to saline treated group (Figure 4) Diaphragm showed increased collagen content in arginine butyrate group (p = 0.004) (Figure 5) Comparison of serum Creatine kinase levels between the groups showed no statistically significant differences (data not shown).

#### Effect on heart function

**Figure 6:**
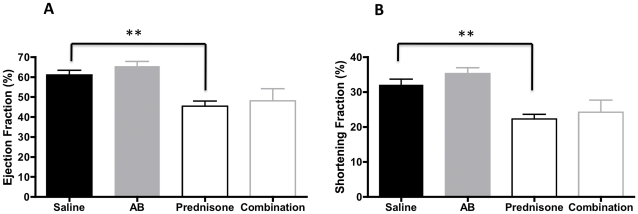
Effect of treatments on cardiac function. Cardiac function was evaluated by echocardiography at the end of the trial. Both the ejection and shortening fractions (%) were evaluated. Error bars indicate +/− 1 SD. **p = <0.01.

At the end of the trial, we evaluated the heart function of the drug-treated and saline control groups by echocardiography. No statistically significant changes occurred in the ejection and shortening fraction of arginine butyrate-treated group (Figure 6). This result is in contrast to the significant decrease in both ejection and shortening fractions that was seen in the prednisone treated group (Figure 6).

#### Effect on utrophin expression

**Figure 7:**
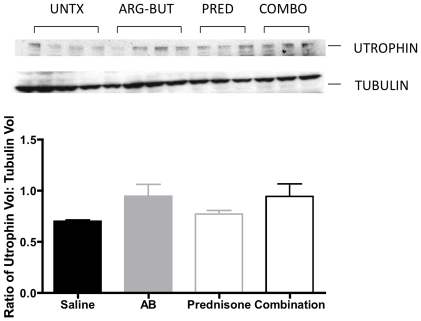
Effect of treatments on utrophin expression in skeletal muscle. Muscle lysates from gastrocnemius muscle were analyzed by Western blotting using anti-utrophin antibody. Beta-tubulin expression was assessed on the same blot as a loading control. The autoradiograms were scanned, and volume analysis was carried out using Quantity One software. The ratios of utrophin to beta-tubulin were calculated for all groups. Error bars indicate +/− 1 SD. **p = <0.01.

Since arginine butyrate is known to induce the expression of fetal genes, including utrophin, we used Western blotting to detect utrophin in skeletal muscle lysates from the drug-treated and saline control groups. We saw variations in the utrophin expression among the arginine butyrate containing groups (Figure 7). Normalization of utrophin expression to tubulin expression, showed a 28% increase in the utrophin expression in the arginine butyrate-treated group and the combination-treated group (Figure 7).

#### Effect on mRNA profiling

**Figure 8:**
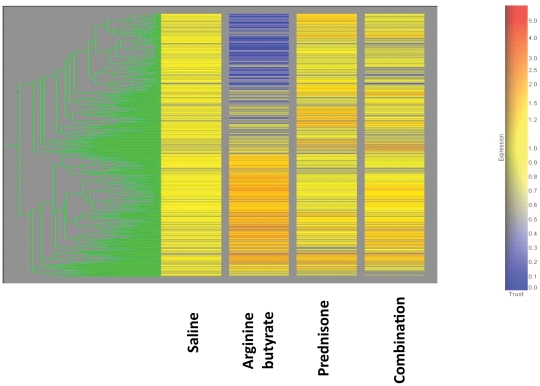
Effect of treatments on gene expression profiling. Temporal clustering of transcriptomes in arginine butyrate-, prednisone- and combination-treated mice and saline-treated controls. Signals were processed using the PLIER algorithm. Unsupervised hierarchical clustering (p<0.01) was done using GeneSpring Software (GeneSpring GX, Fostercity, CA). The data was normalized to the saline treated controls; yellow indicates normal; blue indicated decreased expression and red indicated increased expression of genes.

mRNA expression profiling of gastrocnemius muscle from the drug-treated and saline control groups was performed using Affymetrix gene chips. To interpret the microarray data, we have used multiple probe set algorithms (MAS5.0, dCHIP difference model, PLIER). We have analyzed the data using GeneSpring software. Unsupervised hierarchical clustering showed arginine butyrate treatment drastically altered gene expression in skeletal muscle of dystrophic mice, followed by combination group and prednisone treated groups in comparison to saline treated group (Figure 8). Initially, we looked for genes that showed a two-fold change in expression, with a p-value of 0.01 and the drug treated groups were compared to the saline-treated group. We found that 1515 genes were significantly altered (453 upregulated and 1062 downregulated) in the arginine butyrate-treated group, 136 (72 upregulated and 64 downregulated) in the prednisone-treated group, and 261 (102 upregulated and 159 down regulated) in the combination-treated group. Some of the expressed genes were unique to a particular treatment group (e.g., 1461 genes for arginine butyrate, 103 for prednisone, and 217 for the combination treatment), and others were shared between groups (e.g., the arginine butyrate set shared 20 genes with the prednisone set and 31 genes with the combination set; the combination set shared 10 genes with the prednisone set; 3 genes were shared by all three groups).

We found that genes that participate in multiple cellular activities such as focal adhesion, gap junction, hedgehog signaling pathway, Wnt signaling pathway, calcium signaling pathway, G protein signaling, striated muscle contraction, cell cycle, MAPK signaling pathway, mRNA processing binding reactome, cytokine-cytokine receptor interaction, adipocytokine signaling pathway, insulin signaling pathway, Jak-STAT signaling pathway, mTOR signaling pathway, regulation of actin cytoskeleton, inositol phosphate metabolism, glycan structures – biosynthesis, fatty acid biosynthesis, TGF-beta signaling pathway, PPAR signaling pathway, oxidative phosphorylation, folate biosynthesis, electron transport chain, aminoacyl-tRNA biosynthesis are differentially expressed upon arginine butyrate treatment clearly indicating HDAC inhibitory activity of butyrate [6]. Whereas prednisone affected relatively less number of pathways that control immune functions, cell communication, cytokine-cytokine receptor, Wnt signaling pathway MAPK signaling pathway VEGF signaling pathway, GnRH signaling pathway, arachidonic acid metabolism, biosynthesis of steroids as well as carbohydrate and amino acid metabolism pathways. The combination treated group affected genes that control Wnt signaling pathway, MAPK signaling pathway, regulation of actin cytoskeleton, purine metabolism, Folate biosynthesis, cell communication, ECM-receptor interaction, Sphingolipid metabolism, PPAR signaling pathway, cell cycle, glycan structures – biosynthesis, glycerophospholipid metabolism, SNARE interactions in vesicular transport, and hedgehog signaling pathway. Above pathways are clearly affect disease progression and severity dystrophic skeletal muscle by controlling muscle inflammation, fibrosis, muscle growth and regeneration (supplementary Table S2)

## Discussion

From the time of the discovery and characterization of the gene responsible for Duchenne muscular dystrophy, it was clear that rectifying the primary defect was going to be a challenge. Not only is it by far the largest gene known; too large to be encapsulated in the main viral vectors currently under consideration[7], but also the predominantly affected tissues, skeletal and cardiac muscle cells, are not simple targets. Skeletal muscle, especially, presents the problem of being the most abundant tissue in the body and being so widely and diffusely distributed that the blood vascular system is the only plausible route of delivery of any potentially therapeutic agent. Although both tissues are heavily vascularised, there are no helpful features such as endothelial fenestrae that might provide ready access of blood-borne agents to the muscle fiber that is the target of therapy. While genetic manipulations such as exon skipping have brought us to the verge of therapeutic success in the animal models, none has yet attained a level of efficiency to permit its immediate therapeutic application. In the meantime, there is a need for interim palliative treatments to halt or slow the progress of the disease. Such treatments are aimed at what we perceive to be the pathological pathways downstream of the primary defect, and because we are uncertain of the part played by many of these pathways, or where the optimal balance in e.g. the inflammatory response to muscle necrosis, lies, it is important to take the widest possible view of the benefits and drawbacks of any specific agent and regime of administration. Accordingly, we have developed a protocol that applies a wide range of criteria to evaluation of the effects of specific treatment regimes.

The value of this rationale is well illustrated by the examples of the two agents we have investigated here. One, prednisone is currently ‘standard of care’ for those DMD boys who can tolerate its side effects, but no explanatory mechanism for these benefits has been firmly established. The second agent, arginine butyrate has the potential for beneficial effects via two routes, it makes arginine available as a substrate for nNOS, while the butyrate moiety modifies histones and has widespread effects on gene expression. Here again, the range of potential effector pathways is open to speculation. Our results from evaluating these two agents vindicates our general approach, for we have picked up quite different sets of benefits and dis-benefits from the two treatments, none of which would have been predictable from the existing literature on their biological effects.

In this study, we have taken a comprehensive approach to evaluating the effects of 6 months administration of arginine butyrate, prednisone, and a combination of both drugs on the disease phenotype in *mdx* mice. Our data indicate that 6 months administration of arginine butyrate is well tolerated by the *mdx* mice. We noted a decrease in fibrosis in the skeletal muscle, which correlated well with improvements in grip strength. Furthermore, gene expression profiling of arginine butyrate-treated skeletal muscle indicated an increase in growth-promoting pathways (e.g., the IGF-1 pathway) and a decrease in pro-fibrotic pathways. We did not see any significant differences in behavioral activity, Rotarod performance, histology, or serum CK levels between the drug-treated groups and the saline-treated control group. However, prednisone and combination treated groups showed significant alterations in genes that affect fibrosis, inflammation and myogenesis. This data is consistent with loss of body weight, poor performance on functional and behavioral measures, and decrease in cardiac function and increase in fibrosis.

Since body weight is a simple measure of the overall drug effect on the mouse phenotype, we measured body weight at different times during the trial and found that arginine butyrate had no significant effect on body weight, with arginine butyrate-treated mice showing a growth pattern indistinguishable from that of saline-treated mice. In contrast, prednisone-treated (1 mg/kg/day) with or without combination drug-treated mice showed a significant decrease in body weight. This prednisone-induced decrease in body weight and reduction of weight gain in *mdx* mice has also been noted in previous studies [8], [9]. It appears that normalized soleus mass is increased in all treated groups indicating some degree hypertrophy on the other hand normalized heart mass significantly increased in prednisone and combination treated groups suggesting an increase in cardiomyopathy. Overall these data clearly demonstrate that 6 months administration of arginine butyrate is safe and that chronic continuous administration of prednisone is catabolic to dystrophic muscle and combination is clearly immunosuppressive as reflected by the spleen size. The molecular mechanisms underlying this differential catabolic effect on different tissues and muscle groups are currently being investigated.

Grip strength has been extensively used to evaluate drug effects in the *mdx* mouse model [10], [11], [12], [13], [14], [15]. Absolute forelimb and hindlimb strengths (unadjusted body weight) for the arginine butyrate-treated group at all ages appeared to be greater than those of the other drug treatment groups, suggesting that arginine butyrate treatment improves forelimb and hindlimb strength. Since there was a significant body weight difference between these mice and the prednisone- and combination-treated mice, we normalized the grip strength values to the body weight. Although the arginine butyrate-treated mice did not show a significant difference in body weight, they still showed improved forelimb performance when compared to saline-treated mice. In contrast, after normalization for body weight the prednisone- and combination-treated groups showed significantly better forelimb grip strength because of the decrease in body weight that had occurred with prednisone treatment. In terms of hindlimb grip strength, the prednisone- and combination-treated groups showed a significantly lower level of force than did the arginine butyrate- or saline-treated mice. The reported effects of prednisone on grip strength vary widely, with some showing a significant increase [10], [11], [16] and others reporting no effect [17], [18]. These differences could reflect differences in the ages of the mice, or the doses, routes of administration, and/or duration of prednisone treatment. Our data indicate that there are differences in forelimb and hindlimb grip strength responses and that the prednisone effects are more pronounced in the forelimbs than in the hindlimbs, especially after the results are adjusted for body weight. Our data clearly demonstrate that decrease in normalized forelimb and hindlimb grip strength is stabilized in more in prednisone treated group than arginine butyrate and combination groups.

We also assessed motor coordination using a Rotarod and found that the ability to stay on the Rotarod decreased significantly with age. It appears that none of the drugs we tested had a significant effect on motor coordination under these conditions. We also assessed the overall behavioral activity of the mice using an open field Digiscan apparatus and found that the animals adapted to the Digiscan apparatus over a period of time and generally performed better with time than they did at the initial time point. A comparison of the various treatment groups clearly indicated that the arginine butyrate-treated group generally showed lower horizontal activity, vertical activity, and total distance traversed than did the saline-treated group, but the combination-treated group performed better on all three parameters. The reasons for this poor performance on open field behavioral activity after arginine butyrate treatment are unclear; however, the positive behavioral effects seen with combination group were likely influenced by their reduced body weight when compared to the saline-treated mice.

H&E staining of skeletal muscle of treated mice indicated no significant changes in comparison to saline treated group. It is unclearly why the improvements in grip strength are reflected in improvement in histological changes. Since H&E is not useful for detecting fibrosis, we used Sirius Red, which has been used for many years to detect fibrosis [19]. We found a trend toward a decrease in fibrosis in both the skeletal muscle and heart in arginine butyrate-treated mice. Surprisingly we did not see a decreasing trend in fibrosis of diaphragm in arginine butyrate-treated mice. It is currently unclear how different tissues respond to anti-fibrotic treatments. The trends we observed toward increases in grip strength and cardiac function, together with the decrease in fibrosis after arginine butyrate treatment, indicated a positive effect on skeletal muscle and heart function. Likewise, the opposite trend seen with the prednisone- and combination-treated groups suggests that 6 months treatment with prednisone is detrimental to both skeletal muscle and heart function.

The arginine butyrate treated group have decreased cell size distribution in comparison to saline treated group probably due to increase in trend in fibers with central nuclei/field (regeneration) on the other hand decrease in fiber size is more pronounced in prednisone and combination treated groups correlating with increase in trend in the degenerating fibers on H&E sections.

Our results support the finding that L-arginine-treated *mdx* mice also show an increased coronary flow and reduced diastolic stiffness relative to saline-treated *mdx* mice and that a reduction in stiffness is associated with a significant reduction in cardiac collagen. This suggests that chronic treatment with L-arginine is beneficial in that it improves function and reduces fibrosis in the dystrophin-deficient heart [20]. Furthermore, recent studies have also indicated that L-arginine treatment decreases the activity of NF-kB and metalloproteinases MMP-2 and MMP-9, reduces beta-dystroglycan cleavage, and causes the translocations of utrophin and nNOS throughout the sarcolemma, thus promoting muscle membrane integrity and reducing fibrosis in dystrophic muscle [21]. It is also known that L-arginine treatment blocks macrophage infiltration and the TGF-β_1_-induced collagen deposition seen in tubulointerstitial fibrosis [22], [23]. Recent studies have also indicated that L-arginine treatment significantly reduces necrosis in *mdx* mouse muscle [21], [24], [25]. L- arginine reduces sarcolemmal injury by inducing up-regulation of utrophin and β-dystroglycan levels and increasing the stability of membrane-associated proteins such as integrin-alpha7 [26].

Although arginine butyrate has been reported to induce several fetal genes, we did not see any significant up-regulation of utrophin in the skeletal muscle of arginine butyrate-treated mice at either the protein or the mRNA level. In contrast, L-arginine treatment has previously been shown to induce utrophin expression in *mdx* mouse muscle [25], [27]. The reasons for this discrepancy are unclear; they could be related to the fact that we used arginine butyrate rather than L-arginine. Unlike the results we obtained for arginine butyrate, we saw more pronounced utrophin protein expression in the prednisone- and combination-treated groups than in the other groups. Glucocorticoid treatment of the *mdx* mouse has previously been shown to stimulate utrophin A expression in skeletal muscle fibers [28]. Also, a translational regulatory mechanism involving increased IRES activation has recently been shown to mediate increased expression of utrophin A in muscle cells treated with glucocorticoids [29]. These findings support the notion that one of the mechanisms of glucocorticoid action is through the stimulation of endogenous utrophin levels in dystrophic skeletal muscle fibers [30].

Our results indicated that *mdx* mice generally have higher serum CK levels than control mice and that this was not affected. One published study has indicated that L-arginine treatment reduces serum CK levels [25], and another reported no effect of L-arginine [27] on *mdx* mice. Even though the prednisone- and combination-treated groups showed a trend toward a decrease in serum CK levels, these changes were not statistically significant. Our data regarding prednisone are consistent with the reports beneficial effects on serum CK levels in *mdx* mice [16].

The butyrate moiety of arginine butyrate has been shown to inhibit histone deacetylase, resulting in hyperacetylation of histones H3 and H4. Acetylated histones have a reduced affinity for chromatin, allowing chromosomal unfolding that can potentially modulate the expression of particular genes, including fetal forms of adult genes. This situation is consistent with our observation that 6015 genes were significantly altered in the arginine butyrate-treated mice, 972 in the prednisone-treated mice, and 1411 in the combination-treated mice. Our gene expression profiling data indicate cell proliferation, growth and differentiation as well as genes that control inflammatory, growth-promoting fibrotic genes are differentially affected by arginine butyrate treatment. Genes interacting with collagen deposition, abnormal inflammatory response and degeneration of fibers explain fibrotic effects of prednisone on the skeletal muscle. Likewise combination treated group regulated genes that contribute to muscle wasting and atrophy by inhibiting myogenesis, cell proliferation, interrupting cell cycle and impairing sarcolemmal localization of dystrophin. Further confirmatory experiments are needed to investigate the differentially affected gene pathways at protein level.

Overall, arginine butyrate treatment tended to improve grip strength and decrease fibrosis in the gastrocnemius. These data are supported by the increase in the expression of growth-promoting genes and decrease in pro-fibrotic genes that we observed in the skeletal muscle of arginine butyrate-treated mice. On the other hand, we did not observe significant changes in muscle histology; behavioral measurements, heart function or serum CK levels after arginine butyrate treatment. Detailed experiments need to be carried out in order to evaluate the effects of arginine butyrate treatment in younger mice.

In general, our state understanding of the pathological pathways and our knowledge of the range of possible effects of any given low molecular weight drug on these complex networks of pathways is quite superficial. In such circumstances, it is unwise to constrain analysis to functional effects on the basis of prior expectation based on our limited understanding of the action of these agents in other pathologies. The broad empirical approach we have used here has the merit of increasing the prospects of picking up unexpected beneficial activities. More importantly, such a broad survey also increases the probability of identifying unexpected ill effects such as the increased cardiac fibrosis associated with continuous administration of prednisone. This study highlights the need for comprehensive evaluation strategies to detect both beneficial and harmful effects any drug currently in therapeutic use or for which therapeutic testing is contemplated.

## Supporting Information

Table S1 Comparison of individual muscle and organ weights of treatments in mdx mice.
(0.04 MB DOC)

Table S2 Genes differentially expressed along with fold change.
(0.07 MB PDF)

Figure S1 Effect of 6 months treatment on behavioral assays: A) Rotarod testing. Open field activity (Digiscan)-B) horizontal activity. The combination-treated group (broken grey) showed a smaller increase in horizontal activity than did the arginine butyrate-treated (solid grey), prednisone-treated (broken black), or saline control (solid black) groups. The arginine butyrate-treated group showed less activity than did the rest of the groups. C) Vertical activity. All the groups except the arginine butyrate-treated group showed a progressive increase in their vertical activity. The arginine butyrate-treated group showed a decrease in vertical activity from baseline (3 months of age) to (5 months of age); the activity increased later but never reached that of the other groups. D) Total distance. While the saline-treated group showed a progressive increase in the total distance over the entire 6 months of treatment, the drug-treated groups demonstrated an erratic pattern. The arginine butyrate-treated group showed a decline toward the second month of treatment and recovery when compared to baseline at the end of the trial, with a statistically significant difference from the levels for the prednisone-treated group at 5 months of age. The prednisone-treated group did not change significantly during the trial. The combination-treated group showed an overall increase, with a small reduction at 7 months of age.
(4.29 MB TIF)

Figure S2 Fiber size distribution was evaluated by laminin immunoflourescence and minimal feret measurements. The data were ranked and normalized to sample size (rank number/total sample number). Normalized rank was plotted on the vertical axis against fiber size on the horizontal axis. Treatment groups: saline (blue), arginine butyrate (red), prednisone (green), a combination of arginine butyrate and prednisone (grey).
(3.28 MB TIF)
