# Correction: Functional and Molecular Effects of Arginine Butyrate and Prednisone on Muscle and Heart in the Dystrophin Deficient *mdx* mice

**DOI:** 10.1371/annotation/ccf651e7-2e1d-494d-94cd-bb2fbffff4e7

**Published:** 2010-08-31

**Authors:** Alfredo D. Guerron, Rashmi Rawat, Arpana Sali, Christopher F. Spurney, Emidio Pistilli, Hee-Jae Cha, Gouri S. Pandey, Ramkishore Gernapudi, Dwight Francia, Viken Farajian, Diana M. Escolar, Laura Bossi, Magali Becker, Patricia Zerr, Sabine de la Porte, Heather Gordish-Dressman, Terence Partridge, Eric P. Hoffman, Kanneboyina Nagaraju

A competing interest was omitted. The competing interests section should state that Laura Bossi, Megali Becker, and Patricia Zerr were affiliated with Faust Pharmaceuticals at the time of the study.

