# Correction: Functional and Molecular Effects of Arginine Butyrate and Prednisone on Muscle and Heart in the Dystrophin Deficient *mdx* mice

**DOI:** 10.1371/annotation/a82245dd-7379-4067-bd84-b0fc5ffb5869

**Published:** 2010-08-31

**Authors:** Alfredo D. Guerron, Rashmi Rawat, Arpana Sali, Christopher F. Spurney, Emidio Pistilli, Hee-Jae Cha, Gouri S. Pandey, Ramkishore Gernapudi, Dwight Francia, Viken Farajian, Diana M. Escolar, Laura Bossi, Magali Becker, Patricia Zerr, Sabine de la Porte, Heather Gordish-Dressman, Terence Partridge, Eric P. Hoffman, Kanneboyina Nagaraju

There was an error in the title of the article. The correct title should read: Functional and Molecular Effects of Arginine Butyrate and Prednisone on Muscle and Heart in the Dystrophin Deficient mdx mice. The correct citation is: Guerron AD, Rawat R, Sali A, Spurney CF, Pistilli E, et al. (2010) Functional and Molecular Effects of Arginine Butyrate and Prednisone on Muscle and Heart in the Dystrophin Deficient mdx mice. PLoS ONE 5(6): e11220. doi:10.1371/journal.pone.0011220

