# Correction: Functional and Molecular Effects of Arginine Butyrate and Prednisone on Muscle and Heart in the Dystrophin Deficient *mdx* mice

**DOI:** 10.1371/annotation/a8a69b21-6156-4ea4-9a66-7a5f7410d760

**Published:** 2010-08-31

**Authors:** Alfredo D. Guerron, Rashmi Rawat, Arpana Sali, Christopher F. Spurney, Emidio Pistilli, Hee-Jae Cha, Gouri S. Pandey, Ramkishore Gernapudi, Dwight Francia, Viken Farajian, Diana M. Escolar, Laura Bossi, Magali Becker, Patricia Zerr, Sabine de la Porte, Heather Gordish-Dressman, Terence Partridge, Eric P. Hoffman, Kanneboyina Nagaraju

The affiliation for the 12th, 13th, and 14th authors was incorrect. At the time of the study, Laura Bossi, Magali Becker, and Patricia Zerr were affiliated with Faust Pharmaceuticals SA, Illkirch, France. Magali Becker and Patricia Zerr are currently affiliated with Transgene SA, Illkirch-Graffenstaden, France.

