# Supplementary material for: Functional and Molecular Effects of Arginine Butyrate and Prednisone on Muscle and Heart in the mdx Mouse Model of Duchenne Muscular Dystrophy
Source: PLoS One. 2010 Jun 21;5(6):e11220. doi: 10.1371/journal.pone.0011220 (PMC2888587; doi:10.1371/journal.pone.0011220)
Supplement: Table S1 — Comparison of individual muscle and organ weights of treatments in mdx mice. (0.04 MB DOC) [file pone.0011220.s001.doc]

Table S1 Comparison of individual muscle and organ weights of treatments in mdx mice (absolute and normalized)

| Muscle/Organ | unit | Saline (n=8)* | Arginine-Butyrate (n=8)* | Prednisone (n=7)* | Combination (n=8)* |
| --- | --- | --- | --- | --- | --- |
| Gastrocnemius | (mg) | 143±14.42 | 141.8±12.85 | 119.8±16.18‡ | 114.1±14.45‡ |
|  | (mg/kg) | 5129±274.8 | 5224±411.1 | 5239±743.8 | 4834±364> |
| Soleus | (mg) | 8.6±1.78 | 9.6±1.25‡ | 8.7±1.043 | 8.7±1.5 |
|  | (mg/kg) | 308.2±60.36 | 357±42.75> | 381.4±41.8> | 379.7±52.48> |
| EDL | (mg) | 11.25±3.18 | 12.71±2.34 | 10.99±2.23 | 10.54±1.84 |
|  | (mg/kg) | 404.8±115.1 | 471±63.08 | 481.4±88.41 | 461.8±54.85 |
| Spleen | (mg) | 104.7±8.47 | 87.29±11.95‡ | 85.42±18.78‡ | 68.93±11.94‡ |
|  | (mg/kg) | 3758±246 | 3429±869.3 | 3753±794.5 | 2928±496.1> |
| Heart | (mg) | 111.6±18 | 102.1±17.49 | 105.7±11.32 | 111±16.6 |
|  | (mg/kg) | 3994±497.6 | 3783±601.2 | 4616±482.5> | 4832±616.9> |

*Data presented as mean±SD

‡, > denotes statistical significance in comparison to saline treated group by t-test (p<0.05)
