# Supplementary material for: Functional and Molecular Effects of Arginine Butyrate and Prednisone on Muscle and Heart in the mdx Mouse Model of Duchenne Muscular Dystrophy
Source: PLoS One. 2010 Jun 21;5(6):e11220. doi: 10.1371/journal.pone.0011220 (PMC2888587; doi:10.1371/journal.pone.0011220)
Supplement: Table S2 — Genes differentially expressed along with fold change. (0.07 MB PDF) [file pone.0011220.s002.pdf]

Supplementary Table 2 Genes differentially expressed along with fold change

Arginine Butyrate treated group	Affimetrix Code	Fold Change	Gene
**Upregulated**	1423100_at	6.76	Fos
	1450317_at	6.65	Clec2g
	1420409_at	5.55	Krt35
	1445809_at	5.18	Eya4
	1456593_at	3.87	Sytl2
	1447848_at	3.65	Eps15l1
	1436200_at	3.32	Lonrf3
	1450599_at	3.15	V1rb3
	1431575_at	3.11	Clec4g
	1421728_at	3.03	Olig3
	1419916_at	3.01	Rnf20
	1450687_at	2.95	Igf2bp3
	1456973_at	2.94	Arid5b
	1449681_at	2.91	Hdgf
	1446416_at	2.91	Sulf1
	1456627_at	2.86	Ubqln2
	1422239_at	2.72	Hoxd13
	1459370_at	2.67	Cacng5
	1459381_at	2.50	Dach1
	1426215_at	2.38	Ddc
	1418771_a_at	2.38	Cpb2
	1457113_at	2.38	Nasp
	1436869_at	2.36	Shh
	1419576_at	2.34	Hoxb13
	1422374_s_at	2.33	Olfr64; Olfr66
	1456360_at	2.30	Tes3-ps
	1426080_a_at	2.30	Kcnq2
	1421532_at	2.26	Rxfp2
	1435602_at	2.24	Sephs2
	1446038_at	2.23	Xtrp3s1
	1458396_at	2.16	Ssrp1
	1423424_at	2.16	Zic3
	1448054_at	2.13	Ing4
	1439751_at	2.07	Zfp708
	1460159_at	2.06	Mysm1
	1449736_at	2.04	Pip5k2a
	1435979_a_at	2.02	Myo15b
	1446009_at	2.02	Kcng2
	1457001_at	2.02	Cenpk
**Downregulated**	1418189_s_at	-47.39	Malat1
	1424922_a_at	-44.64	Brd4
	1418188_a_at	-40.16	Malat1
	1427353_at	-38.91	Clasp1
	1437001_at	-32.79	Gsk3b
	1416501_at	-28.57	Pdpk1
	1427742_a_at	-27.40	Klf6

	1427511_at	-25.64	B2m
	1427262_at	-24.10	Xist
	1436983_at	-23.47	Crebbp
	1458089_at	-22.17	Fkbp5
	1438736_at	-19.57	Thoc2
	1449168_a_at	-18.73	Akap2
	1426805_at	-18.69	Smarca4
	1437417_s_at	-17.76	Gpc6
	1438186_at	-17.61	Pdlim5
	1423432_at	-17.45	Phip
	1447930_at	-16.29	Baz1a
	1459791_at	-15.58	Dnajc1
	1439970_at	-14.75	Wnk1
	1428936_at	-14.41	Atp2b1
	1437106_at	-14.06	Jarid1a
	1444083_at	-13.95	Ttn
	1443702_at	-13.89	Mtap4
	1420816_at	-13.53	Ywhag
	1457817_at	-13.37	Bcas3
	1438401_at	-13.12	Ubn1
	1427665_a_at	-12.36	Nfic
	1430529_at	-12.32	Csnk1a1
	1424398_at	-12.17	Dhx36
	1459068_at	-12.09	Tmem109
	1447504_at	-12.03	Ghitm
	1440609_at	-11.60	Map4k4
	1444647_at	-11.56	Plaa
	1421604_a_at	-11.52	Klf3
	1453240_a_at	-11.52	Gcap14
	1441823_at	-11.48	Zmiz1
	1457689_at	-11.47	Sbf2
	1444194_at	-11.27	Mllt10
	1417084_at	-11.04	Eif4ebp2
	1455831_at	-10.91	Fus
	1418562_at	-10.56	Sf3b1
	1445337_at	-10.45	Dnajc13
	1439972_at	-10.32	Etnk1
	1445147_at	-10.29	Psme4
	1417705_at	-10.28	Otub1
	1446332_at	-10.27	Pcdhga12
	1450661_x_at	-10.26	Nfic
	1439572_at	-10.24	R3hdm1
	1452236_at	-10.09	Abcf1
	1460336_at	-9.52	Ppargc1a
	1459463_at	-9.52	Nptn
	1446272_at	-9.43	Pctk2
	1426951_at	-9.43	Crim1
	1441561_at	-9.26	Fbxl3
	1426587_a_at	-9.17	Stat3

	1460086_at	-9.17	Mnab
	1457198_at	-9.09	Nrp1
	1437158_at	-9.09	Nipbl
	1425538_x_at	-9.09	Ceacam1
	1452690_at	-9.01	Khsrp
	1419038_a_at	-9.01	Csnk2a1
	1457491_at	-8.93	Plekha1
	1416801_at	-8.62	Trpm7
	1429464_at	-8.62	Prkaa2
	1425050_at	-8.62	Isoc1
	1431739_at	-8.55	Mto1
	1436952_at	-8.55	Klf9
	1421907_at	-8.47	Pparbp
	1427486_at	-8.40	Ptprb
	1438663_at	-8.40	Bat2d
	1443057_at	-8.33	Sos2
	1438271_at	-8.33	Lpp
	1427037_at	-8.33	Eif4g1
	1436362_x_at	-8.26	Ccrn4l
	1457731_at	-8.20	Snapc3
	1417248_at	-8.20	Ralbp1
	1445866_at	-8.13	Mast4
	1456677_at	-8.13	Herc4
	1419283_s_at	-7.87	Tns1
	1443602_at	-7.81	Ifngr1
	1438207_at	-7.81	Gbf1
	1448579_at	-7.58	Glg1
	1443603_at	-7.52	Pcmtd1
	1438807_at	-7.46	Hnrpr
	1457343_at	-7.41	Ap4s1
	1429487_at	-7.35	Ppp1r12a
	1437821_at	-7.35	Diap1
	1446861_at	-7.30	Gns
	1443220_at	-7.25	Rtn3
	1423597_at	-7.25	Atp8a1
	1441050_at	-7.25	Ap4s1
	1440247_at	-7.19	Phf14
	1455886_at	-7.19	Cbl
	1446943_at	-7.14	Dst
	1450295_s_at	-7.09	Pvr
	1438268_at	-6.99	Mnab
	1441297_at	-6.90	Muc6
	1415859_at	-6.90	Eif3s8
	1421398_at	-6.85	Trim7
	1420372_at	-6.80	Sntb2
	1454875_a_at	-6.76	Rbbp4
	1439168_at	-6.71	Camk2d
	1444066_at	-6.67	Gapvd1
	1416568_a_at	-6.62	Acin1

	1445720_at	-6.54	Sltm
	1447038_x_at	-6.49	Phip
	1460328_at	-6.49	Brd3
	1437657_at	-6.45	Zfp291
	1420422_at	-6.45	Pcdhb21
	1439108_at	-6.45	Mll5
	1451270_at	-6.45	Dusp18
	1422910_s_at	-6.41	Smc6
	1453094_at	-6.41	Ches1
	1454665_at	-6.37	Irf2bp2
	1447649_x_at	-6.37	Dnajc1
	1459237_at	-6.37	Atf2
	1442102_at	-6.29	Polr3h
	1457297_at	-6.29	Mef2a
	1425587_a_at	-6.25	Ptprj
	1447770_at	-6.25	Cib2
	1450434_s_at	-6.21	Pcyt1a
	1444503_at	-6.21	Gbas
	1437892_at	-6.17	Zfp306
	1440092_at	-6.17	Ext1
	1457511_at	-6.13	Rgs12
	1458176_at	-6.13	Per3
	1457264_at	-6.10	Phf20l1
	1434020_at	-6.10	Pdap1
	1421957_a_at	-6.10	Pcyt1a
	1427797_s_at	-6.10	Ctse
	1427228_at	-6.06	Palld
	1449042_at	-6.06	Ctcf
	1426541_a_at	-6.02	Endod1
	1457630_at	-5.99	Ppapdc1
	1443522_s_at	-5.99	Phip
	1439272_at	-5.99	Lcorl
	1449101_at	-5.99	Ebf2
	1448155_at	-5.92	Pdcd6ip
	1450747_at	-5.92	Keap1
	1419156_at	-5.88	Sox4
	1429003_at	-5.85	Snw1
	1439532_s_at	-5.85	Kif13a
	1428174_x_at	-5.85	Khsrp
	1441209_at	-5.78	Rin2
	1443897_at	-5.78	Ddit3
	1440104_at	-5.75	Ranbp2
	1417472_at	-5.75	Myh9
	1438508_at	-5.75	Jmjd1b
	1442453_at	-5.75	Fcho2
	1430037_at	-5.71	Snx27
	1422272_at	-5.71	Phxr4
	1446095_at	-5.71	Air
	1446464_at	-5.59	Psme4

	1430369_at	-5.59	Epb4.1
	1446481_at	-5.59	Apbb2
	1428343_at	-5.56	Rcor3
	1437700_at	-5.52	Schip1
	1429463_at	-5.49	Prkaa2
	1444114_at	-5.46	Mkln1
	1450177_at	-5.43	Ngfr
	1452158_at	-5.43	Eprs
	1438700_at	-5.41	Fnbp4
	1442395_at	-5.41	Dst
	1429624_at	-5.38	Sltm
	1420610_at	-5.35	Prkacb
	1432344_a_at	-5.35	Aplp2
	1460717_at	-5.29	Tspyl1
	1437446_at	-5.29	Rab5b
	1429779_at	-5.29	Eif2c4
	1441117_at	-5.29	Ash1l
	1442632_at	-5.26	Centg2
	1438131_at	-5.24	Fbxw2
	1450093_s_at	-5.21	Zbtb7a
	1420170_at	-5.21	Myh9
	1458233_at	-5.21	Fryl
	1437222_x_at	-5.18	Rrm2b
	1441185_at	-5.18	Msi2
	1420946_at	-5.18	Atrx
	1449089_at	-5.13	Nrip1
	1431828_a_at	-5.08	Synj2
	1424504_at	-5.05	Rab22a
	1440543_at	-5.05	Heatr5a
	1425610_s_at	-5.05	Galnt2
	1438258_at	-5.00	Vldlr
	1452885_at	-5.00	Sfrs2ip
	1431028_a_at	-5.00	Pank1
	1449311_at	-5.00	Bach1
	1439248_at	-4.98	Rmi1
	1460393_a_at	-4.95	Dusp7
	1441265_at	-4.93	Plekhm2
	1425461_at	-4.93	Fbxw11
	1454030_at	-4.88	Saps3
	1458532_at	-4.88	Mtr
	1456505_at	-4.88	Braf
	1442878_at	-4.85	Prdx6
	1444810_at	-4.85	Acaca
	1442277_at	-4.83	Chka
	1460215_at	-4.81	Rpo1-4
	1426777_a_at	-4.78	Wasl
	1428497_at	-4.78	Secisbp2
	1428130_at	-4.76	Lman1
	1437789_at	-4.76	Birc6

	1450341_at	-4.74	Pcdhb8
	1421453_at	-4.74	Jph2
	1459371_at	-4.74	Eif4e
	1458994_at	-4.74	Csnk1g3
	1445317_at	-4.72	Sdha
	1425362_at	-4.72	Hrbl
	1447109_at	-4.72	Elavl1
	1426366_at	-4.67	Eif2c2
	1453224_at	-4.63	Zfand5
	1456880_at	-4.63	Hpvc2
	1421890_at	-4.61	St3gal2
	1447018_at	-4.59	Pum1
	1441946_at	-4.57	Itih5
	1436533_at	-4.55	Trove2
	1436257_at	-4.52	Ss18
	1450656_at	-4.52	Gna13
	1449945_at	-4.50	Ppargc1b
	1458242_at	-4.50	Gphn
	1427652_x_at	-4.46	Synj2
	1444295_at	-4.46	Neo1
	1429427_s_at	-4.42	Tcf7l2
	1422842_at	-4.39	Xrn2
	1420867_at	-4.39	Tmed2
	1421915_a_at	-4.39	St3gal3
	1441466_at	-4.33	Sfrs10
	1441230_at	-4.33	Fndc3b
	1418285_at	-4.31	Efnb1
	1419891_s_at	-4.31	C77545
	1417820_at	-4.27	Tor1b
	1456131_x_at	-4.26	Dag1
	1449999_a_at	-4.26	Cacna2d1
	1457744_at	-4.22	Ddx46
	1419806_at	-4.20	Hdlbp
	1425457_a_at	-4.18	Grb10
	1438824_at	-4.15	Slc20a1
	1424112_at	-4.15	Igf2r
	1455876_at	-4.12	Slc4a7
	1442158_at	-4.12	Mast4
	1416497_at	-4.10	Pdia4
	1444604_at	-4.10	Ezh1
	1417310_at	-4.08	Tob2
	1442697_at	-4.08	Ipo11
	1454200_at	-4.07	Zfhx1b
	1441573_at	-4.07	Scmh1
	1454664_a_at	-4.07	Eif5
	1424658_at	-4.05	Taok1
	1452497_a_at	-4.03	Nfatc3
	1437545_at	-4.02	Rcor1
	1423872_a_at	-4.02	Dag1

	1437926_at	-4.00	Xpnpep3
	1425932_a_at	-4.00	Cugbp1
	1439773_at	-3.95	Ly6e
	1429298_at	-3.95	Ddah1
	1416504_at	-3.92	Ulk1
	1430418_at	-3.92	Tmem57
	1430932_at	-3.92	Slc9a8
	1418245_a_at	-3.92	Rbm9
	1437842_at	-3.92	Plcxd1
	1449931_at	-3.92	Cpeb4
	1429338_a_at	-3.91	Nol9
	1442442_at	-3.89	Thoc7
	1416724_x_at	-3.88	Tcf4
	1439598_at	-3.88	Foxo3a
	1445322_x_at	-3.86	Sqle
	1437477_at	-3.85	Lrrfip1
	1456043_at	-3.83	Usp22
	1440441_at	-3.80	Fbxl7
	1458408_at	-3.79	Samd8
	1450515_at	-3.79	Kcnj11
	1426756_at	-3.79	Galnt2
	1437479_x_at	-3.77	Tbx3
	1458069_at	-3.77	Tbc1d5
	1424503_at	-3.77	Rab22a
	1422095_a_at	-3.76	Tyki
	1450184_s_at	-3.76	Tef
	1441526_at	-3.76	Mbtd1
	1423250_a_at	-3.75	Tgfb2
	1432151_at	-3.73	Gtpbp9
	1438806_at	-3.73	Bcl9l
	1425535_at	-3.72	Repin1
	1456382_at	-3.72	Atad1
	1417961_a_at	-3.70	Trim30
	1425444_a_at	-3.70	Tgfbr2
	1453164_a_at	-3.70	Ptdss2
	1427950_at	-3.69	Zfp294
	1443911_at	-3.68	Xpo1
	1421205_at	-3.68	Atm
	1458637_x_at	-3.66	Ube3c
	1447164_at	-3.65	Rlf
	1421268_at	-3.64	Ugcg
	1429449_at	-3.64	Samd4
	1448541_at	-3.61	Kns2
	1422738_at	-3.61	Ddr2
	1441869_x_at	-3.61	Auts2
	1419099_x_at	-3.57	Stom
	1447475_at	-3.57	Gm967
	1448776_at	-3.57	Gadd45gip1
	1452742_at	-3.53	Trak1

	1438739_at	-3.53	Cnbp
	1442623_at	-3.50	Mef2a
	1446068_at	-3.50	Adk
	1419155_a_at	-3.46	Sox4
	1460035_at	-3.46	Phb2
	1440298_at	-3.45	Treml2
	1458039_at	-3.45	Ncoa3
	1448965_at	-3.45	Inoc1
	1440736_at	-3.45	AI131651
	1421011_at	-3.44	Hsd17b11
	1439922_at	-3.41	Prrc1
	1452267_at	-3.41	Flywch1
	1426226_at	-3.41	Dyrk1a
	1443086_at	-3.40	Alcam
	1456054_a_at	-3.36	Pum1
	1444952_a_at	-3.36	Nucks1
	1449069_at	-3.34	Zfp148
	1423117_at	-3.34	Pum1
	1454015_a_at	-3.32	Cdh13
	1442316_x_at	-3.31	Trp53bp1
	1431060_at	-3.31	Peli1
	1446006_at	-3.31	Dph4
	1418849_x_at	-3.28	Aqp7
	1433574_at	-3.27	Cdc37l1
	1421721_a_at	-3.27	Arnt
	1421324_a_at	-3.23	Akt2
	1450458_at	-3.22	Ncoa2
	1431755_a_at	-3.21	Ccdc49
	1421922_at	-3.19	Sh3bp5
	1456256_at	-3.19	Eif5
	1451638_s_at	-3.19	Armc1
	1457455_at	-3.18	Suhw4
	1421323_a_at	-3.18	G3bp2
	1440844_at	-3.17	Tob1
	1456667_at	-3.17	Hdh
	1439314_at	-3.16	Clock
	1421961_a_at	-3.15	Dnajb5
	1457812_at	-3.14	Trp53bp1
	1433896_at	-3.14	Tmem127
	1442993_at	-3.14	Itgb3bp
	1459542_at	-3.11	Prkg1
	1448456_at	-3.09	Cln8
	1419584_at	-3.08	Ttc28
	1448023_at	-3.08	Kalrn
	1418290_a_at	-3.08	Ezh1
	1421718_at	-3.06	Strm
	1438674_a_at	-3.06	Sfrs8
	1450650_at	-3.06	Myo10
	1427478_at	-3.03	Usp12

	1453487_at	-3.03	Dhdh
	1440751_at	-3.01	Ep400
	1417209_at	-3.00	Sertad2
	1436714_at	-3.00	Lpp
	1427764_a_at	-2.99	Tcfe2a
	1429415_at	-2.99	Prkcbp1
	1424442_a_at	-2.99	Pja2
	1429145_at	-2.99	Nhlrc2
	1445827_at	-2.98	Prkcbp1
	1453498_x_at	-2.96	Steap3
	1440579_at	-2.96	Mib1
	1418714_at	-2.96	Dusp8
	1425161_a_at	-2.94	Trabd
	1451887_at	-2.93	Lrba
	1459734_at	-2.92	Psmd14
	1453099_at	-2.92	Csnk2a2
	1458526_at	-2.91	Rpgrip1
	1460386_a_at	-2.90	Slc1a1
	1424077_at	-2.89	Gdpd1
	1439079_a_at	-2.89	Erbb2ip
	1450253_a_at	-2.88	Map3k4
	1457402_at	-2.87	Sulf1
	1457639_at	-2.87	Atp6v1h
	1416403_at	-2.87	Abcb10
	1423065_at	-2.87	Dnmt3a
	1458078_at	-2.86	Chd9
	1425800_at	-2.85	Rad9b
	1436763_a_at	-2.83	Klf9
	1430131_at	-2.83	Crry
	1435216_a_at	-2.82	Odf2
	1415798_at	-2.82	Ddr1
	1460191_at	-2.81	Ykt6
	1424997_at	-2.81	Sfrs8
	1452259_at	-2.81	Phf20
	1437667_a_at	-2.81	Bach2
	1430328_at	-2.80	Polr3f
	1458508_at	-2.80	Matr3
	1437784_at	-2.79	Runx1t1
	1440464_at	-2.79	Elavl1
	1421895_at	-2.78	Eif2s3x
	1445116_at	-2.77	Usp25
	1424459_at	-2.77	Aytl2
	1427199_at	-2.76	Fryl
	1457704_at	-2.75	Zfp533
	1440013_at	-2.75	Trim44
	1429428_at	-2.75	Tcf7l2
	1431068_at	-2.75	Rmnd5a
	1419484_a_at	-2.75	Gbas
	1430704_at	-2.74	Irak3

	1450439_at	-2.73	Hcfc1
	1420836_at	-2.72	Slc25a30
	1446743_at	-2.72	Las1l
	1429517_at	-2.72	Zfyve20
	1427967_at	-2.72	Srgap2
	1437554_at	-2.72	Plec1
	1425611_a_at	-2.71	Cutl1
	1428877_at	-2.68	Srp72
	1440573_at	-2.68	Erbb2ip
	1431024_a_at	-2.68	Arid4b
	1431062_a_at	-2.67	Exoc4
	1442909_at	-2.67	Kcnq4
	1458480_at	-2.62	Topbp1
	1427741_x_at	-2.62	Nkx2-3
	1451251_at	-2.61	Appbp2
	1451494_at	-2.60	Wac
	1440039_at	-2.60	Brwd3
	1422102_a_at	-2.58	Stat5b
	1459307_at	-2.57	Sec24b
	1421047_at	-2.56	Smad5
	1418612_at	-2.56	Slfn1
	1425940_a_at	-2.56	Ssbp3
	1437149_at	-2.54	Slc6a6
	1450018_s_at	-2.54	Slc25a30
	1426061_x_at	-2.54	Cul4a
	1426472_at	-2.52	Zfp52
	1438516_at	-2.52	Rif1
	1442760_x_at	-2.51	Rtn4
	1442038_at	-2.51	Rbm26
	1459844_at	-2.51	Tnip2
	1444557_at	-2.50	Sumo1
	1424134_at	-2.50	Rspry1
	1431230_a_at	-2.50	Btbd9
	1451020_at	-2.49	Gsk3b
	1416081_at	-2.48	Smad1
	1445059_at	-2.48	C80435
	1418628_at	-2.47	Khdrbs1
	1442528_at	-2.46	Lats2; Xpo4
	1444562_at	-2.44	Ube3c
	1439966_x_at	-2.44	Sfxn2
	1458985_at	-2.44	Fry
	1453427_at	-2.44	Csnk2a1
	1449445_x_at	-2.43	Mfap1a
	1453559_a_at	-2.40	Sel1l
	1423066_at	-2.40	Dnmt3a
	1438539_at	-2.40	Ascc1
	1456952_at	-2.38	Arid1b
	1453313_at	-2.38	Sesn3
	1447206_at	-2.35	Arhgap21

	1441612_at	-2.33	Zfp148
	1439233_at	-2.33	Tloc1
	1441611_at	-2.32	Zdhhc6
	1425526_a_at	-2.31	Prrx1
	1426164_a_at	-2.30	Usf1
	1439196_at	-2.30	Hook3
	1419370_a_at	-2.29	Mfap1a
	1426076_at	-2.29	Cdc27
	1460237_at	-2.29	Trim8
	1421145_at	-2.29	Slc26a2
	1443568_x_at	-2.29	Pacs1
	1419191_at	-2.29	Hipk3
	1423456_at	-2.29	Bzw2
	1427574_s_at	-2.28	Sh3d19
	1441628_at	-2.27	Diap3
	1440551_at	-2.27	Dnajc1
	1453681_at	-2.27	Atpif1
	1457834_at	-2.26	Yy1
	1458142_at	-2.26	Zdhhc9
	1456997_at	-2.26	Ncoa5
	1441038_at	-2.24	Utrn
	1457183_at	-2.23	Slc6a1
	1422399_a_at	-2.23	Rab23
	1440747_at	-2.23	Gpr15
	1440610_at	-2.23	Casd1
	1423522_at	-2.23	Npm3
	1430355_a_at	-2.22	Steap3
	1446295_at	-2.22	Trim24
	1449515_at	-2.21	Zfp292
	1426993_at	-2.21	Xpr1
	1441548_at	-2.20	Frmd4b
	1420893_a_at	-2.20	Tgfbr1
	1439650_at	-2.20	Rtn4
	1453684_s_at	-2.19	Zc3h15
	1421002_at	-2.19	Angptl2
	1417131_at	-2.18	Cdc25a
	1421896_at	-2.18	Elk1
	1450902_at	-2.17	Brd3
	1430058_at	-2.16	Slbp
	1430560_at	-2.16	Ppp1r10
	1427607_at	-2.16	Cacna1h
	1446323_at	-2.16	Atf7ip
	1424886_at	-2.15	Ptprd
	1446529_at	-2.15	AI449175
	1425605_a_at	-2.14	Lmbr1
	1455677_s_at	-2.14	Clcnka
	1423202_a_at	-2.13	Ncor1
	1422607_at	-2.13	Etv1
	1425702_a_at	-2.13	Enpp5

	1440935_at	-2.13	Grb10
	1427117_at	-2.12	Mtmr3
	1440450_at	-2.12	Hspa1l
	1436577_at	-2.12	Arhgef9
	1453888_at	-2.11	Cpne4
	1457158_at	-2.10	Setd6
	1449876_at	-2.10	Prkg1
	1441767_at	-2.09	Zfp142
	1447988_at	-2.09	Pdzd2
	1460653_at	-2.08	Atxn2
	1421024_at	-2.08	Agpat1
	1436470_at	-2.07	Rims2
	1425459_at	-2.07	Mtmr2
	1455586_at	-2.07	Rnf168
	1439639_at	-2.07	Phc2
	1447931_at	-2.06	Whsc1l1
	1422811_at	-2.06	Slc27a1
	1457540_at	-2.06	Slc25a26
	1416493_at	-2.04	Ddost
	1456705_at	-2.04	Zfp532
	1422820_at	-2.04	Lipe
	1423120_at	-2.04	Ide
	1434602_at	-2.03	Thrap2
	1430777_a_at	-2.02	Golph3
	1453315_at	-2.01	Wibg
**Arginine-Butyrate/Prednisone treated group**	**Affimetrix Code**	**Fold Change**	**Gene**
**Upregulated**	1420801_at	8.439	Npas1
	1445942_at	5.826	AU015858
	1452892_at	5.204	Stk33
	1417556_at	4.402	Fabp1
	1443599_at	3.987	Dcst1
	1421572_at	2.933	Hif3a
	1456684_at	2.566	Tmem74
	1436200_at	2.558	Lonrf3
	1447622_at	2.513	Fcgr2b
	1418686_at	2.489	Oas1c
	1427789_s_at	2.455	Gnas
	1444688_at	2.394	Pcqap
	1420782_at	2.365	Tnfrsf17
	1455865_at	2.355	Insm1
	1422210_at	2.247	Foxd3
	1434583_at	2.087	Tmem26
**Downregulated**	1449299_at	-6.06	Lrp5
	1424673_at	-4.59	Clec2h
	1456239_at	-4.29	Fgf17
	1459317_at	-4.24	Ank2
	1416033_at	-3.79	Tmem109
	1430829_s_at	-3.46	MGI:1347093
	1449160_at	-3.08	Npr1

	1448776_at	-3.06	Gadd45gip1
	1420230_at	-3.03	AA414993
	1417975_at	-2.91	Kpna4
	1443370_at	-2.82	Ascc3
	1420988_at	-2.75	Polh
	1449956_at	-2.44	Prkce
	1425111_at	-2.44	Sorcs3
	1452793_at	-2.42	Dzip1
	1427099_at	-2.34	Maz
	1418153_at	-2.31	Lama1
	1456305_x_at	-2.29	Obox1; Obox5
	1455575_at	-2.24	Eif4ebp2
	1434944_at	-2.19	Dmpk
	1421709_a_at	-2.18	Fmo5
	1433967_at	-2.18	LOC433492
	1452690_at	-2.17	Khsrp
	1430059_at	-2.12	Wdr40a
	1456927_at	-2.09	Mast2
	1458035_at	-2.08	Sgpp2
	1426027_a_at	-2.00	Arhgap10
**Prednisone treated group**	**Affimetrix Code**	**Fold Change**	**Gene**
**Upregulated**	1425034_at	5.45	Slc17a2
	1450575_at	3.98	Chrm4
	1422143_at	3.90	Akap7
	1416063_x_at	3.37	Ceacam11
	1420409_at	3.09	Krt35
	1420782_at	2.27	Tnfrsf17
	1453711_at	2.25	Rspo4
	1429651_at	2.18	Phactr3
	1445573_at	2.05	Ppp1r14b
	1422210_at	2.04	Foxd3
	1456679_at	2.01	Obfc1
	1446416_at	2.01	Sulf1
**Downregulated**	1440891_at	-4.42	Gria4
	1459116_at	-3.89	Ncam2
	1426730_a_at	-3.68	Prlpk
	1415969_s_at	-3.60	Kap
	1442437_at	-3.51	Ptk2b
	1425790_a_at	-3.38	Grik2
	1422350_at	-2.25	Fpr-rs3
	1456723_at	-2.14	Zfp689
	1447504_at	-2.11	Ghitm
	1437601_at	-2.07	Otx1
	1439600_at	-2.04	Nadk

Genes expressed in gastrocemius muscle (p <0.01). Riken sequences have been removed

;

l. Cut off +/- 2.
