# Supplementary figures and images for: Functional and Molecular Effects of Arginine Butyrate and Prednisone on Muscle and Heart in the mdx Mouse Model of Duchenne Muscular Dystrophy
Source: PLoS One. 2010 Jun 21;5(6):e11220. doi: 10.1371/journal.pone.0011220 (PMC2888587; doi:10.1371/journal.pone.0011220)

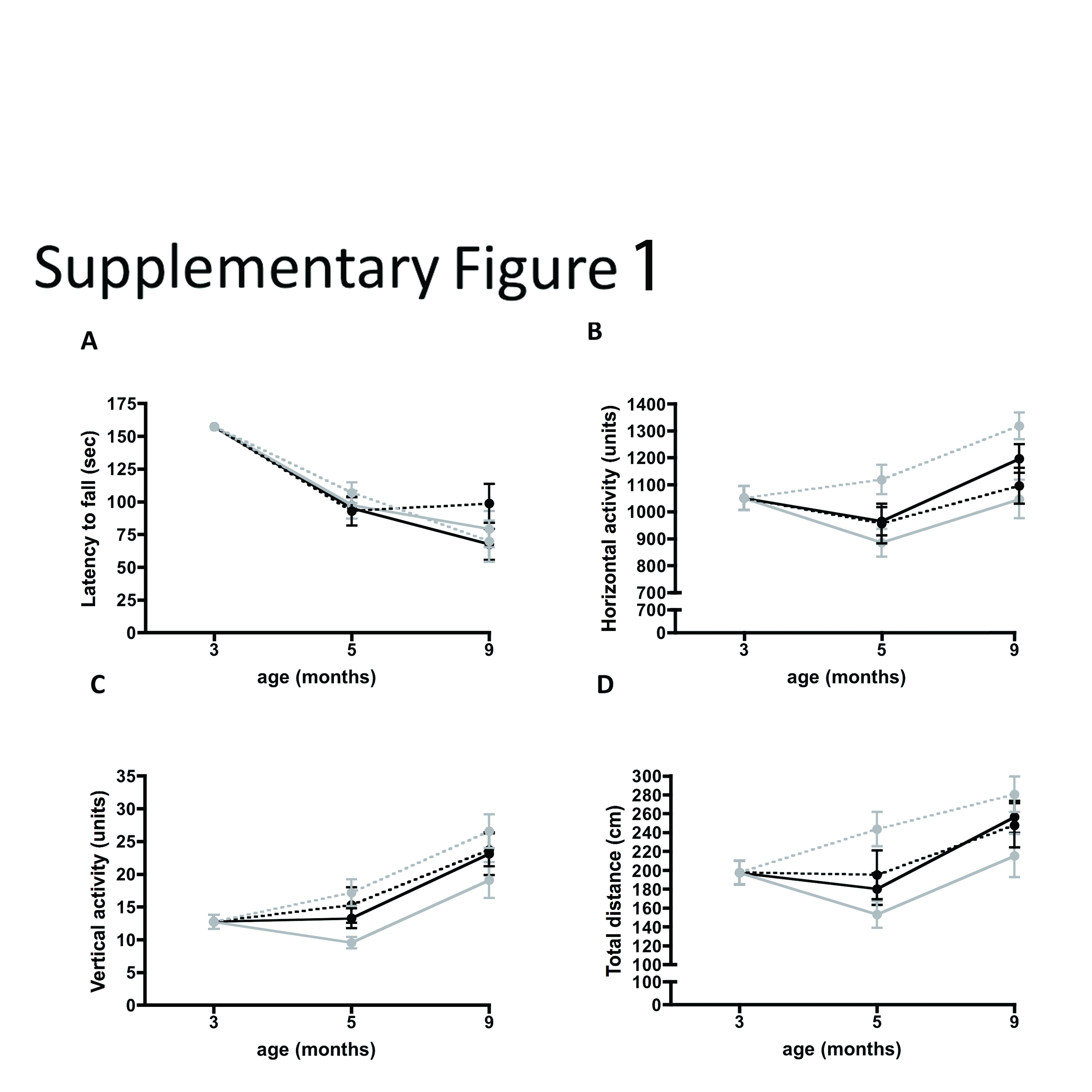

Supplement: Figure S1 — Effect of 6 months treatment on behavioral assays: A) Rotarod testing. Open field activity (Digiscan)-B) horizontal activity. The combination-treated group (broken grey) showed a smaller increase in horizontal activity than did the arginine butyrate-treated (solid grey), prednisone-treated (broken black), or saline control (solid black) groups. The arginine butyrate-treated group showed less activity than did the rest of the groups. C) Vertical activity. All the groups except the arginine butyrate-treated group showed a progressive increase in their vertical activity. The arginine butyrate-treated group showed a decrease in vertical activity from baseline (3 months of age) to (5 months of age); the activity increased later but never reached that of the other groups. D) Total distance. While the saline-treated group showed a progressive increase in the total distance over the entire 6 months of treatment, the drug-treated groups demonstrated an erratic pattern. The arginine butyrate-treated group showed a decline toward the second month of treatment and recovery when compared to baseline at the end of the trial, with a statistically significant difference from the levels for the prednisone-treated group at 5 months of age. The prednisone-treated group did not change significantly during the trial. The combination-treated group showed an overall increase, with a small reduction at 7 months of age. (4.29 MB TIF) [file pone.0011220.s003.tif]

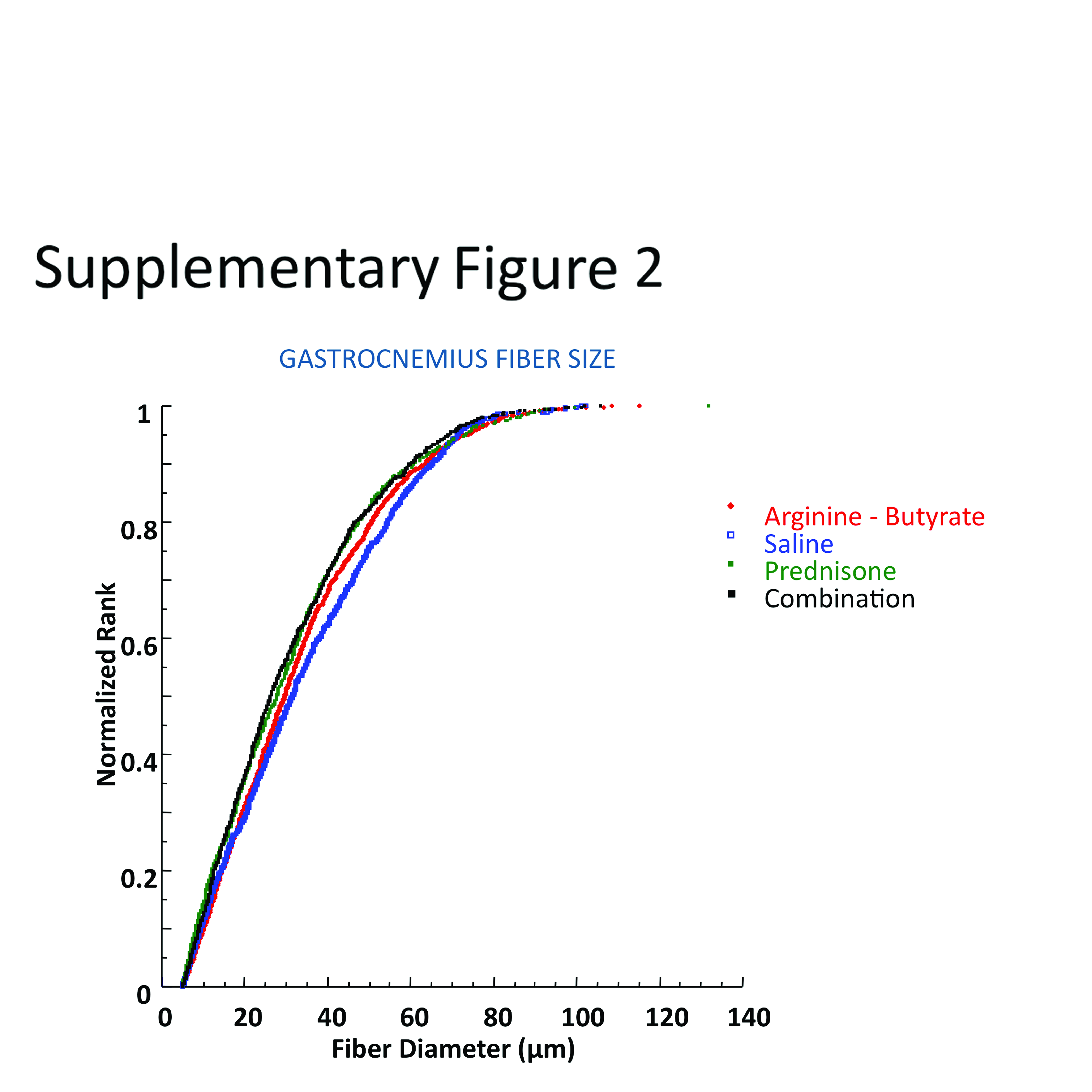

Supplement: Figure S2 — Fiber size distribution was evaluated by laminin immunoflourescence and minimal feret measurements. The data were ranked and normalized to sample size (rank number/total sample number). Normalized rank was plotted on the vertical axis against fiber size on the horizontal axis. Treatment groups: saline (blue), arginine butyrate (red), prednisone (green), a combination of arginine butyrate and prednisone (grey). (3.28 MB TIF) [file pone.0011220.s004.tif]
